# Supplementary material for: A model-free method for genealogical inference without phasing and its application for topology weighting
Source: Genetics. 2025 Sep 8;232(1):iyaf181. doi: 10.1093/genetics/iyaf181 (PMC12774849; doi:10.1093/genetics/iyaf181)
Supplement: iyaf181_Supplementary_Data [file iyaf181_supplementary_data.zip › Supplementary_Figure_1_GENETICS-2025-308408.pdf]

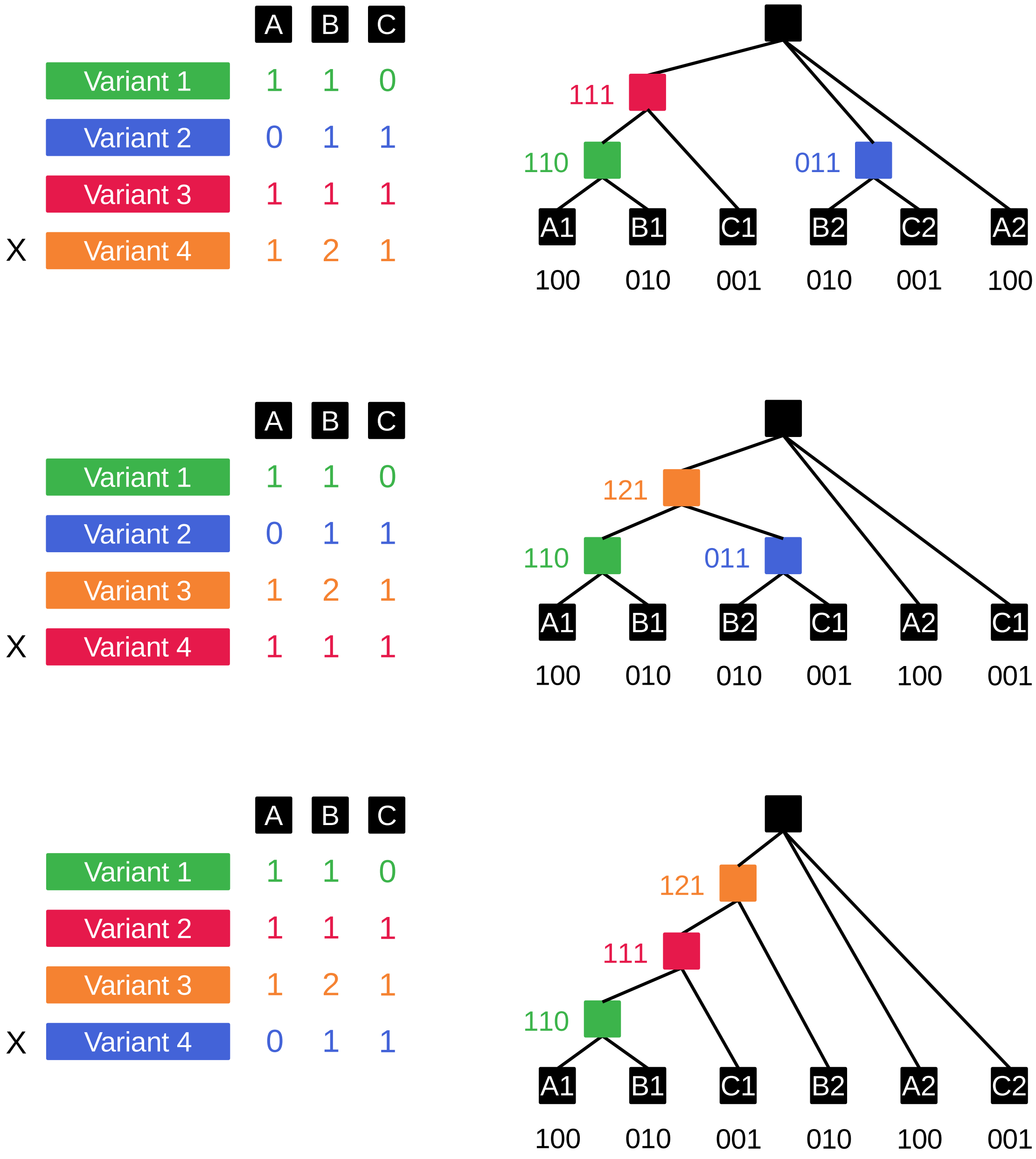

Supplementary Figure 1. Examples of conflicts when applying perfect phylogeny tree building to diploid genotypes. *Four variant patterns for three diploid individuals are shown, in three different orderings. All pairs of variant patterns are mutually compatible according to the generalised four gamete test described in the main text. Nevertheless, the four variant patterns are not compatible with a single tree. Applying the ordered tree building procedure shown in Figure 1 of the main text results in a different final tree depending on the order in which variant patterns are added. In in each case one pattern is discarded (indicated by 'X') because no suitable child nodes can be found.*
