# Supplementary material for: A model-free method for genealogical inference without phasing and its application for topology weighting
Source: Genetics. 2025 Sep 8;232(1):iyaf181. doi: 10.1093/genetics/iyaf181 (PMC12774849; doi:10.1093/genetics/iyaf181)
Supplement: iyaf181_Supplementary_Data [file iyaf181_supplementary_data.zip › Supplementary_Figure_2_GENETICS-2025-308408.pdf]

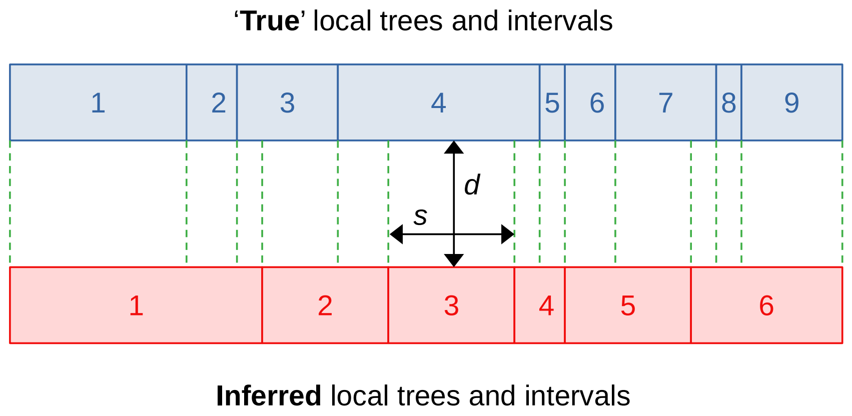

$$D = \frac{\sum^{n} d_i s_i}{\sum^{n} s_i}$$

Supplementary Figure 2. Diagram showing how ARG inference accuracy is quantified. *The ARG can be represented as a series of local trees separated by breakpoints. Here each numbered box indicates a different local tree and its span on the chromosome. Inferred ARGs can differ from the true underlying ARG not only in their tree topologies, but also in the number of trees and the locations of breakpoints. To compare topologies in an inferred ARG (red) to the truth (blue), we therefore first need to decide which trees to compare with which. This is done by dividing the chromosome at every breakpoint in both ARGs (dashed lines). This creates a new set of n intervals. Each of the n intervals overlaps exactly one tree in the true ARG and one tree in the inferred ARG. We therefore compute n distances (d), one for each of the n intervals. To get the overall distance (D) we take a weighted mean of the n values of d, weighted by the relative span (s) of each interval.*
