# Supplementary material for: A model-free method for genealogical inference without phasing and its application for topology weighting
Source: Genetics. 2025 Sep 8;232(1):iyaf181. doi: 10.1093/genetics/iyaf181 (PMC12774849; doi:10.1093/genetics/iyaf181)
Supplement: iyaf181_Supplementary_Data [file iyaf181_supplementary_data.zip › Supplementary_Figure_3_GENETICS-2025-308408.pdf]

**A. Infinite sites mutation**, rho/theta=1, no gene conv., no errors, no pop. struct.

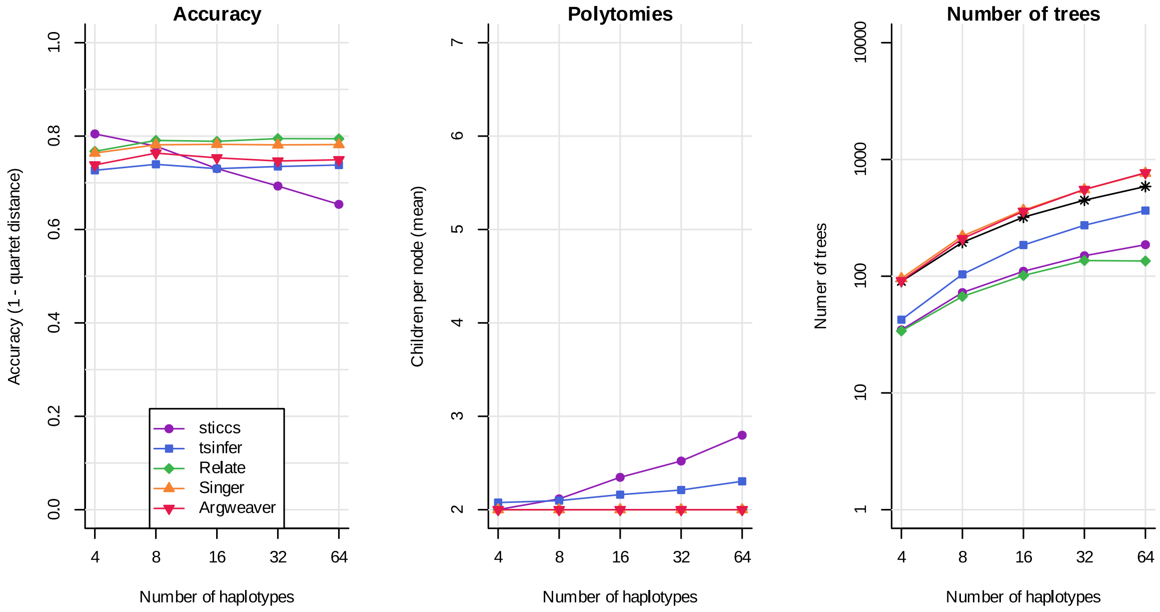


**B. Finite sites mutation**, rho/theta=1, no gene conv., no errors, no pop. struct.

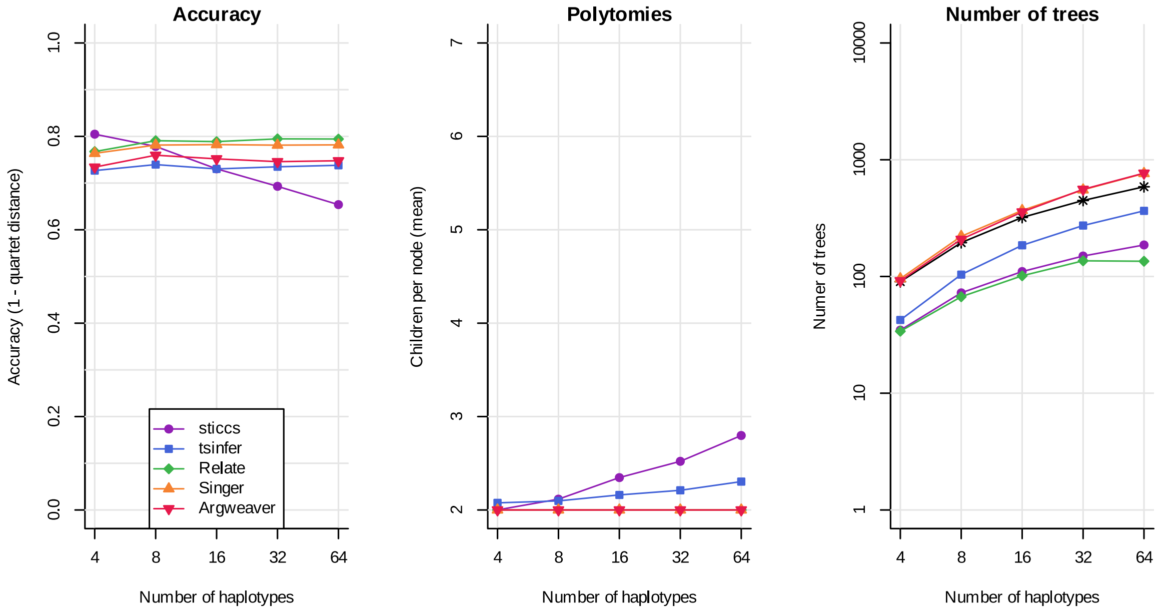


***Supplementary Figure 3 (Panels A and B). Assessment of ARG inference accuracy.*** *Left panel shows the accuracy of local tree topology and breakpoint inference using sticcs and four other popular ARG inference tools. The x-axis indicates the number of haplotypes analysed. Middle panel shows the mean number of children per node per tree, with deviations above 2.0 indicating the presence of unresolved nodes (polytomies). Note that only sticcs and tsinfer allow polytomies. Right panel shown the total number of trees in the 100kb ARG inferred using each method, with the simulated true number shown with black astrices. All plotted results are averaged over 20 simulations of a 100kb region.* ***Panel A****: infinite sites mutational model, $N_e$=1e5, mu=1e-8, r=1e-8.* ***Panel B****: Finite sites mutational model, $N_e$=1e5, mu=1e-8, r=1e-8.*

**C.** Finite sites mutation, **rho/theta=10**, no gene conv., no errors, no pop. struct.

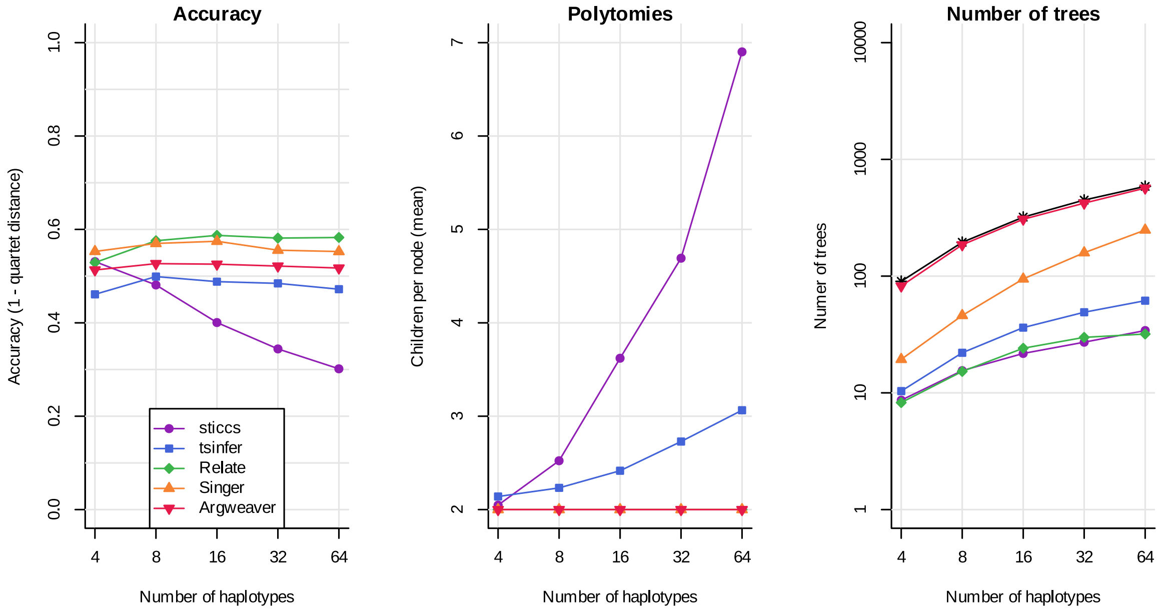


**D.** Finite sites mutation, rho/theta=1, **with gene conv.**, no errors, no pop. struct.

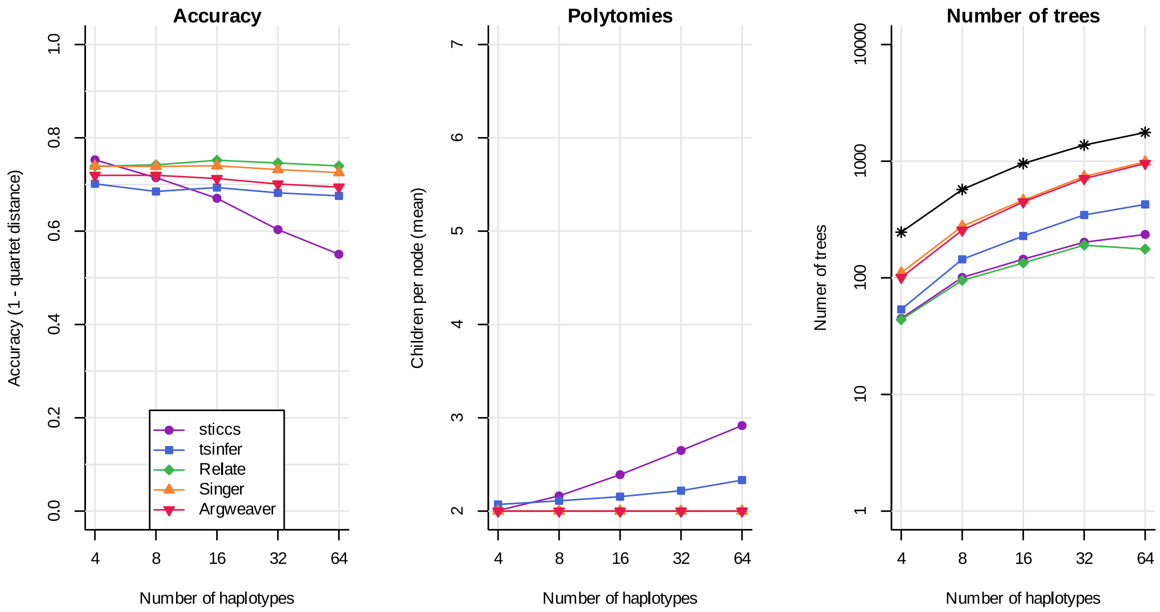


***Supplementary Figure 3 (panels C and D). Assessment of ARG inference accuracy.*** *Left panel shows the accuracy of local tree topology and breakpoint inference using sticcs and four other popular ARG inference tools. The x-axis indicates the number of haplotypes analysed. Middle panel shows the mean number of children per node per tree, with deviations above 2.0 indicating the presence of unresolved nodes (polytomies). Note that only sticcs and tsinfer allow polytomies. Right panel shown the total number of trees in the 100kb ARG inferred using each method, with the simulated true number shown with black astrices. All plotted results are averaged over 20 simulations of a 100kb region.* ***Panel C****: with high recombination rate, $N_e$=1e5, mu=1e-9, r=1e-8.* ***Panel D****: with gene conversion, $N_e$=1e5, mu=1e-8, r=1e-8, GC rate=1e-8, GC mean tract length=300.*

**E.** Finite sites mutation, rho/theta=1, no gene conv., **with genotyping errors**, no pop. struct.

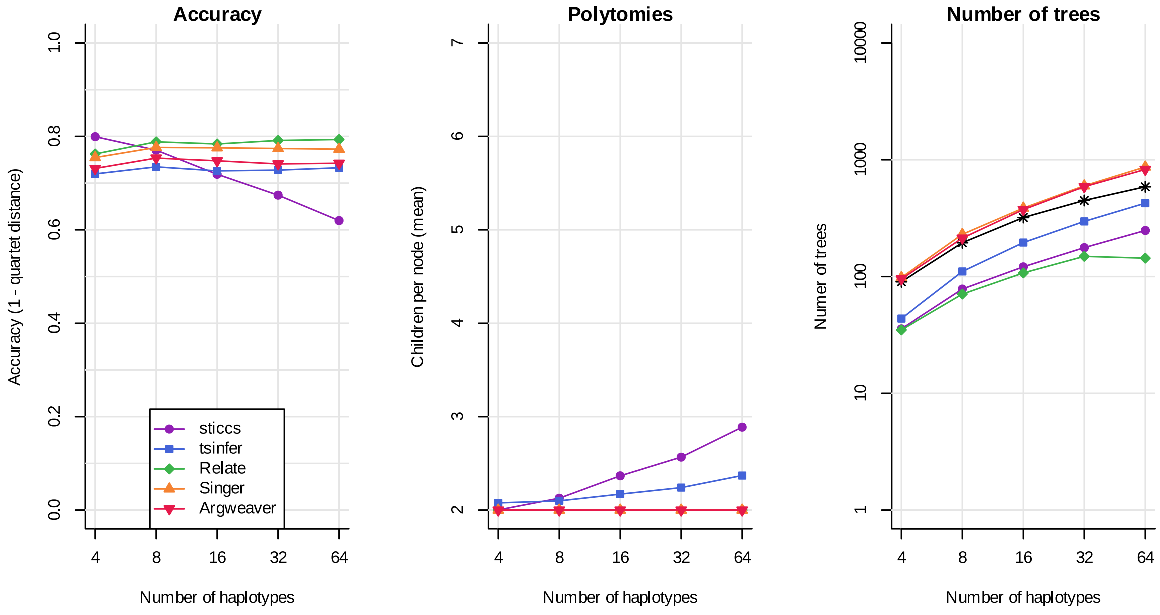


**F.** Finite sites mutation, rho/theta=1, with gene conv., **with polarization errors**, no pop. struct.

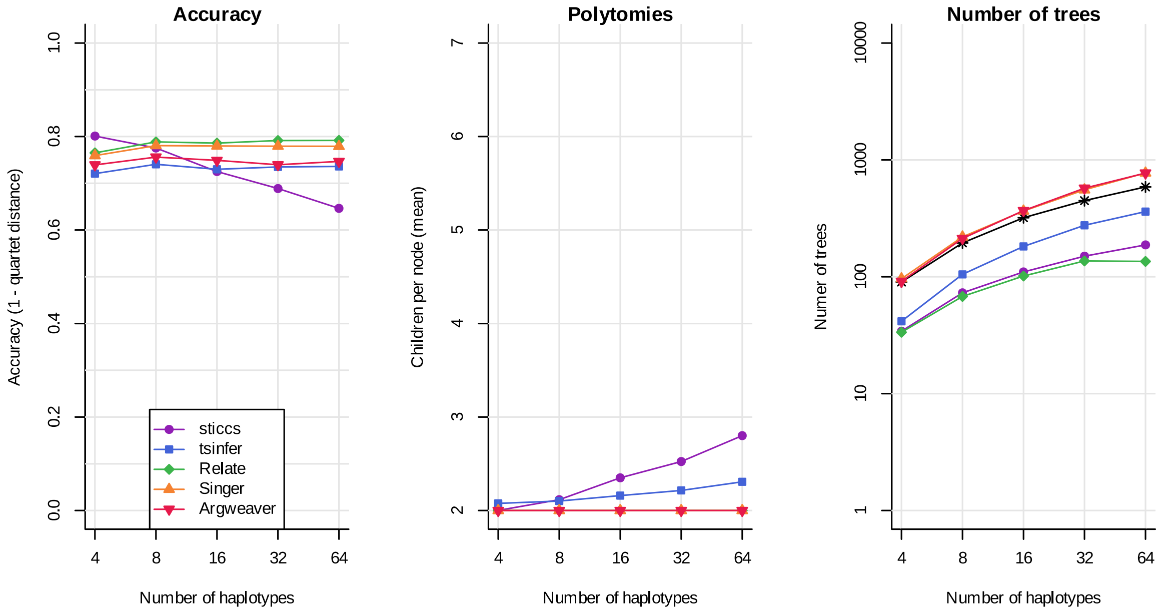


***Supplementary Figure 3 (panels E and F). Assessment of ARG inference accuracy.*** *Left panel shows the accuracy of local tree topology and breakpoint inference using sticcs and four other popular ARG inference tools. The x-axis indicates the number of haplotypes analysed. Middle panel shows the mean number of children per node per tree, with deviations above 2.0 indicating the presence of unresolved nodes (polytomies). Note that only sticcs and tsinfer allow polytomies. Right panel shown the total number of trees in the 100kb ARG inferred using each method, with the simulated true number shown with black astrices. All plotted results are averaged over 20 simulations of a 100kb region.* ***Panel E****: with genotyping errors,* $N_e$*=1e5, mu=1e-8, r=1e-8.* ***Panel F****: with polarisation errors,* $N_e$*=1e5, mu=1e-8, r=1e-8.*

**G.** Finite sites mutation, rho/theta=1, no gene conv., no errors, **with pop. struct. (Fst ~ 0.5)**

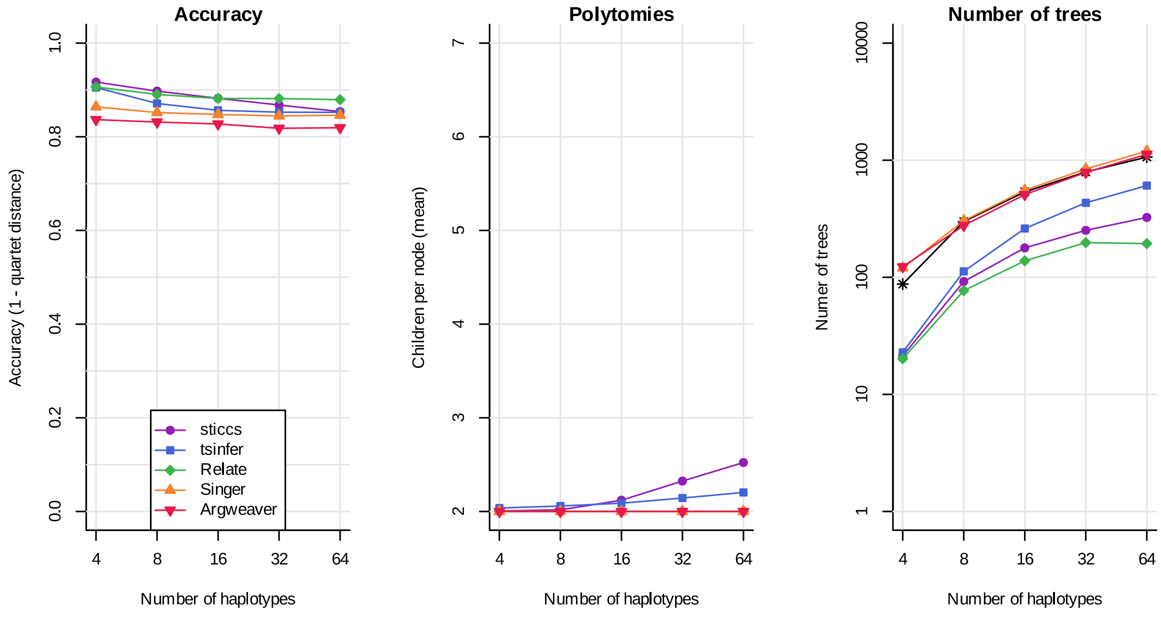


***Supplementary Figure 3 (panel G). Assessment of ARG inference accuracy.*** *Left panel shows the accuracy of local tree topology and breakpoint inference using sticcs and four other popular ARG inference tools. The x-axis indicates the number of haplotypes analysed. Middle panel shows the mean number of children per node per tree, with deviations above 2.0 indicating the presence of unresolved nodes (polytomies). Note that only sticcs and tsinfer allow polytomies. Right panel shown the total number of trees in the 100kb ARG inferred using each method, with the simulated true number shown with black astrices. All plotted results are averaged over 20 simulations of a 100kb region.* ***Panel G****: with population structure, $N_e$=1e5, mu=1e-8, r=1e-8, split time=2N generations ago.*
