# Supplementary material for: A model-free method for genealogical inference without phasing and its application for topology weighting
Source: Genetics. 2025 Sep 8;232(1):iyaf181. doi: 10.1093/genetics/iyaf181 (PMC12774849; doi:10.1093/genetics/iyaf181)
Supplement: iyaf181_Supplementary_Data [file iyaf181_supplementary_data.zip › Supplementary_Figure_4_GENETICS-2025-308408.pdf]

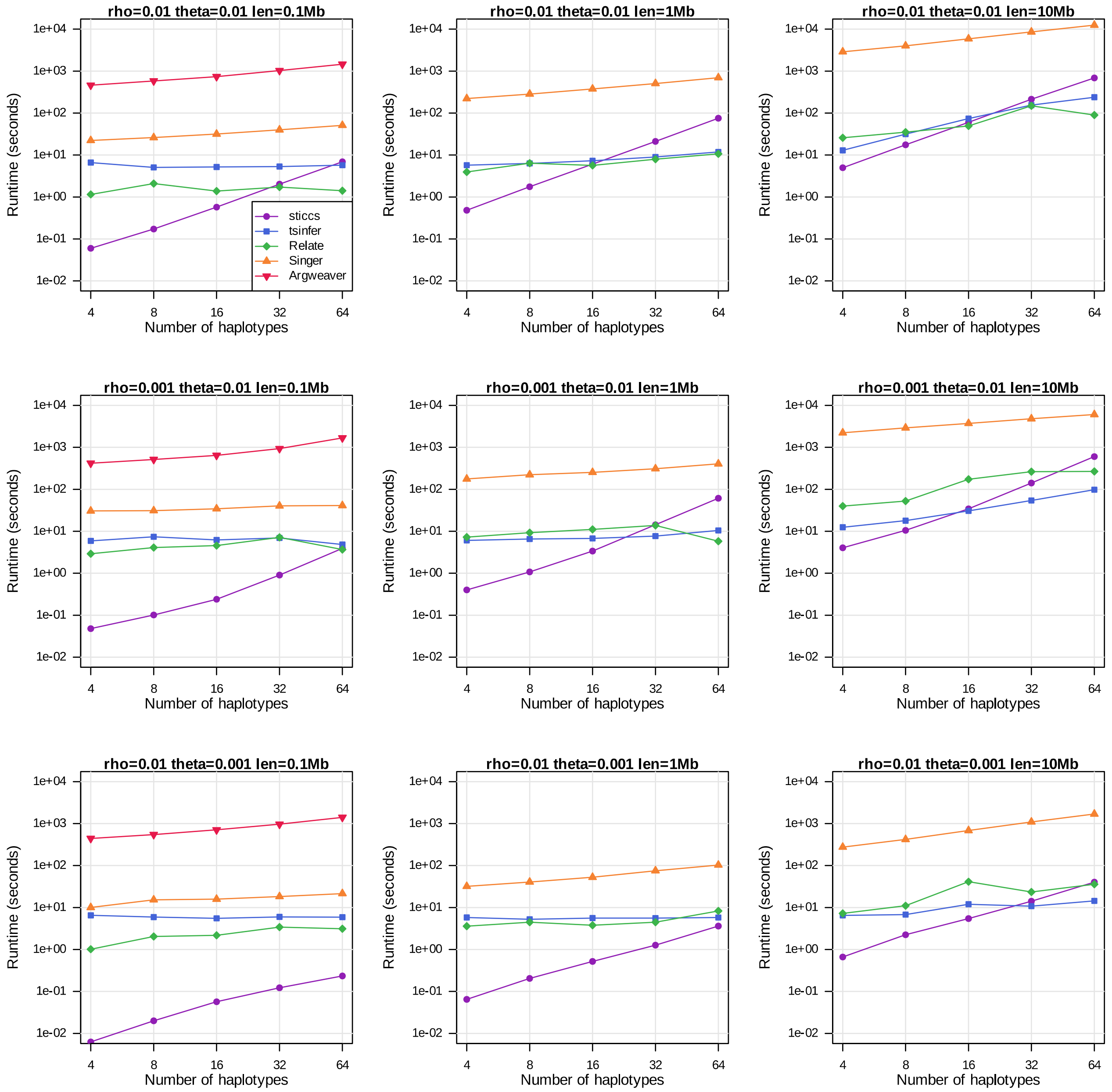

Supplementary Figure 4. Runtime comparisons. *Each panel shows the runtime of each method for a given rho=$N_e r$, theta=$N_e \mu$ and sequence length. The runtime of sticcs is nearly quadratic with sample size. It is linear with sequence length (equivalent points in graphs of the same row jump by an order of magnitude as the sequence is increased by the same amount). Runtime is slowest when theta is high (first two rows), and does not depend much on rho. ARGweaver was only tested at the 0.1Mb scale due to its slow runtime.*
