# Supplementary material for: A model-free method for genealogical inference without phasing and its application for topology weighting
Source: Genetics. 2025 Sep 8;232(1):iyaf181. doi: 10.1093/genetics/iyaf181 (PMC12774849; doi:10.1093/genetics/iyaf181)
Supplement: iyaf181_Supplementary_Data [file iyaf181_supplementary_data.zip › Supplementary_Figure_5_GENETICS-2025-308408.pdf]

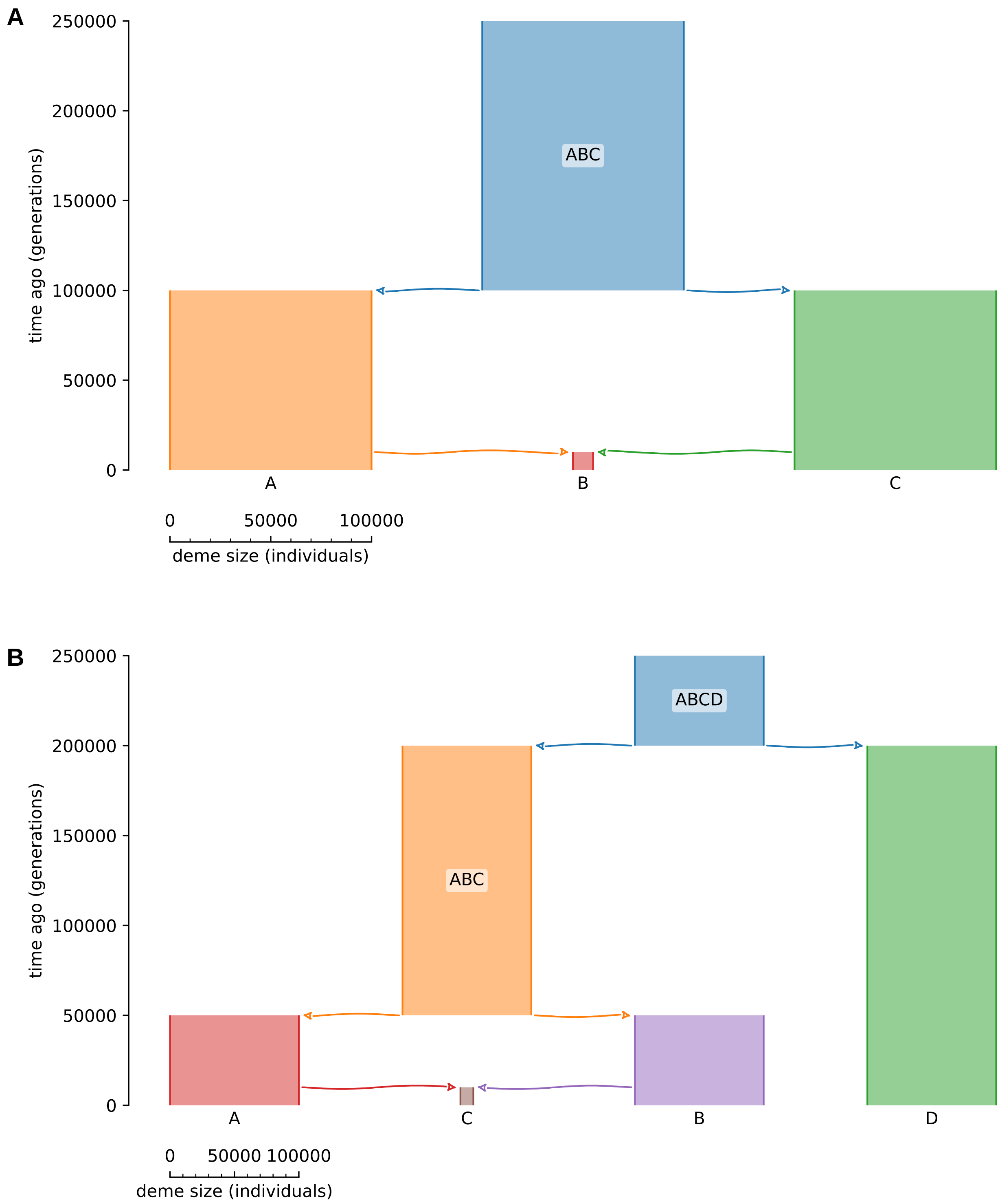

Supplementary Figure 5. Simulation models for topology weighting tests. Panel A. *Three-species admixture scenario.* ***Panel B.*** *Four-species admixture scenario. A small recipient population was used because this causes large fluctuations in ancestry along the genome without the need to simulate selection. Equal proportions of ancestry from both parental species was simulated.*
