# Supplementary material for: A model-free method for genealogical inference without phasing and its application for topology weighting
Source: Genetics. 2025 Sep 8;232(1):iyaf181. doi: 10.1093/genetics/iyaf181 (PMC12774849; doi:10.1093/genetics/iyaf181)
Supplement: iyaf181_Supplementary_Data [file iyaf181_supplementary_data.zip › Supplementary_Figure_6_GENETICS-2025-308408.pdf]

A. Three-species admixture scenario

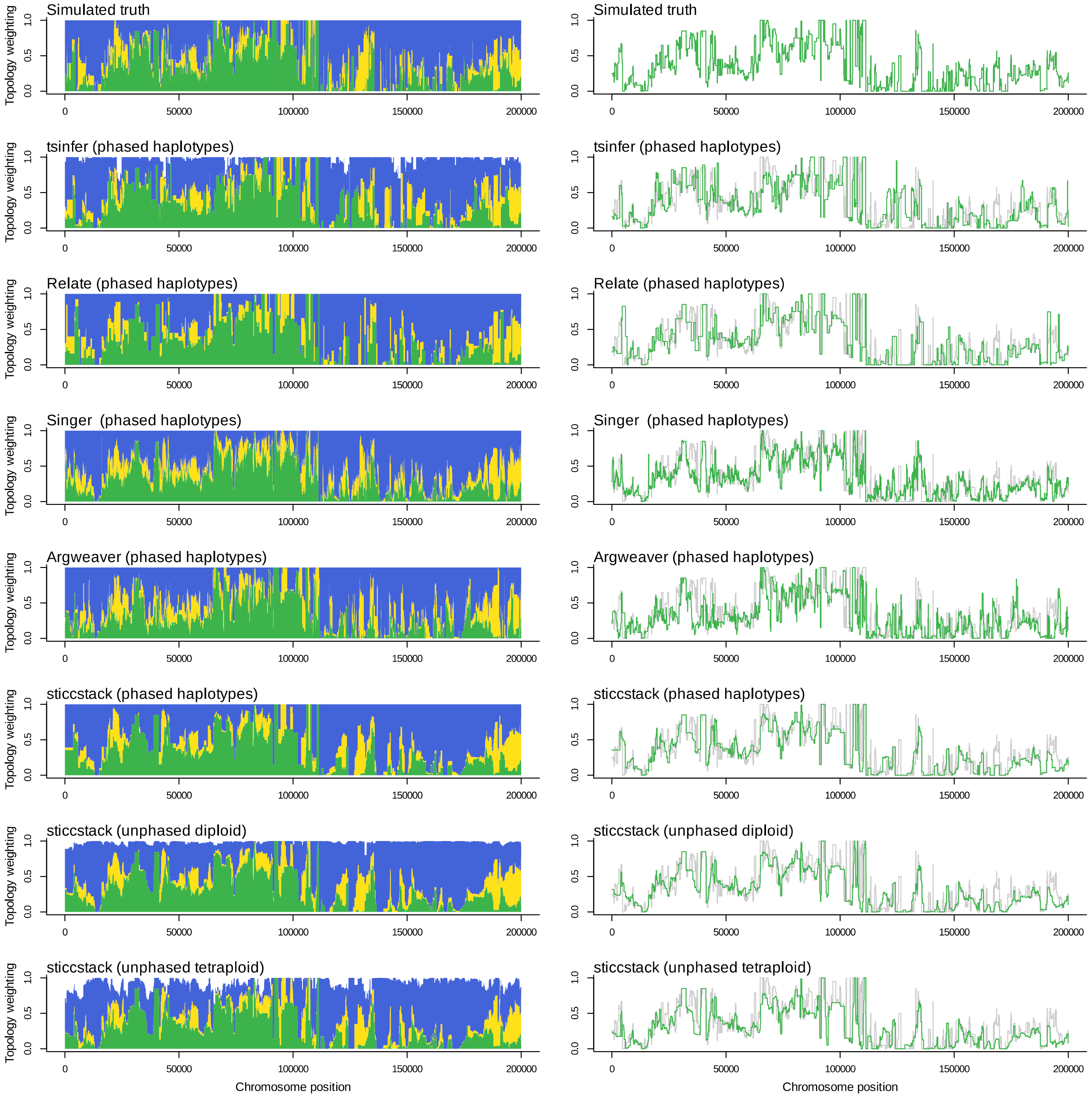

Supplementary Figure 6 (Panel A). Visual comparison of topology weights inferred using different methods. *Simulations were performed under the three-species admixture scenario (Panel A in Supplementary Figure 5). The top row shows the simulated truth. Subsequent rows show weightings computed using inferred ARGs using different methods. Left panels show weightings for all three subtree topologies. Right panel shows weightings for one of the topologies plotted over the simulate truth in grey.*

B. Four-species admixture scenario

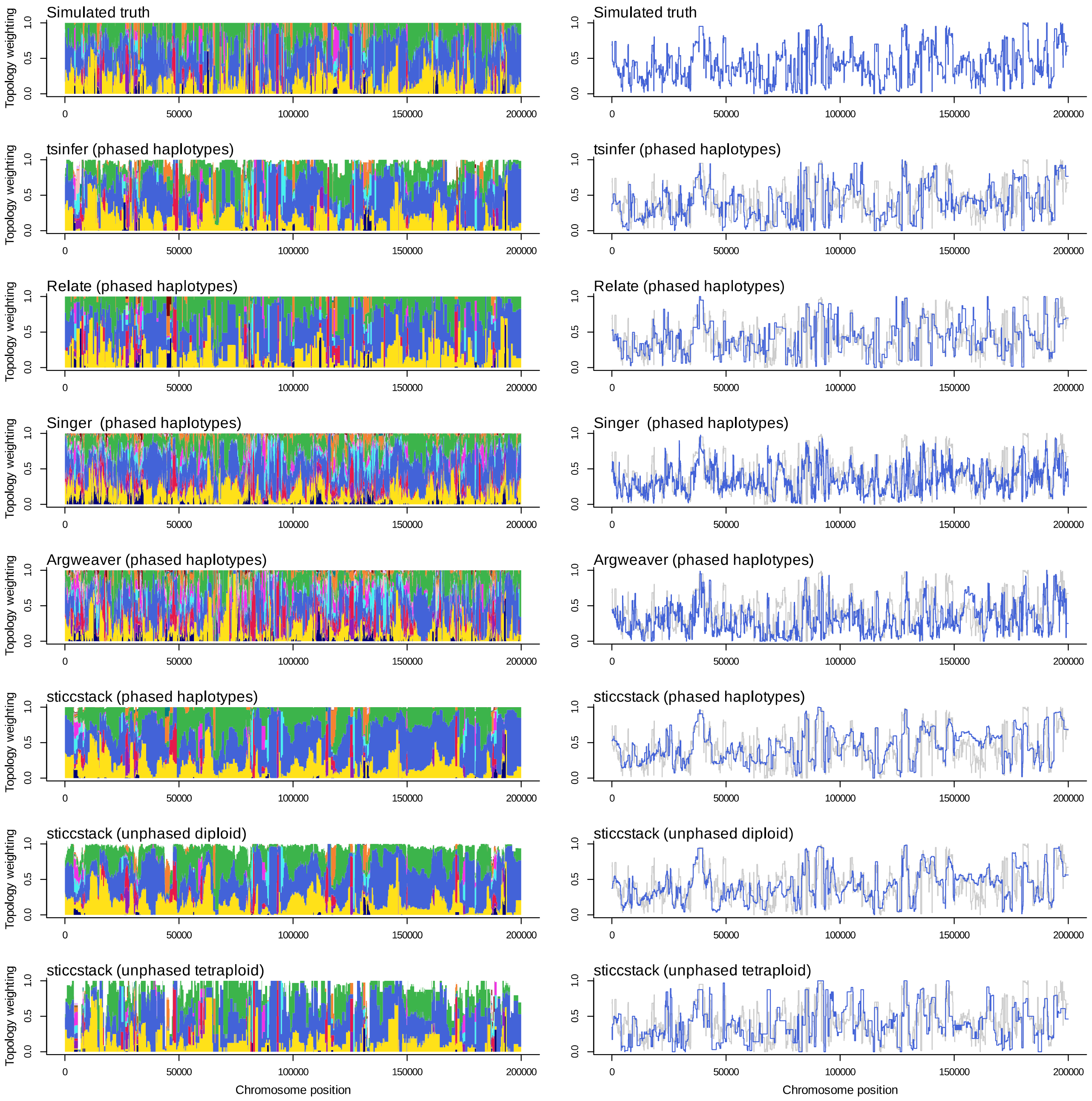

Supplementary Figure 6 (Panel B). Visual comparison of topology weights inferred using different methods. *Simulations were performed under the four-species admixture scenario (Panel B in Supplementary Figure 5). The top row shows the simulated truth. Subsequent rows show weightings computed using inferred ARGs using different methods. Left panels show weightings for all three subtree topologies. Right panel shows weightings for one of the topologies plotted over the simulate truth in grey.*
